# Computed tomography with low-dose radiation versus standard-dose radiation for diagnosing fractures: systematic review and meta-analysis

**DOI:** 10.1590/1516-3180.2020.0374.R3.1902021

**Published:** 2021-06-14

**Authors:** Márcio Luís Duarte, Lucas Ribeiro dos Santos, Acary Souza Bulle Oliveira, Wagner Iared, Maria Stella Peccin

**Affiliations:** I MD, MSc. Musculoskeletal Radiologist, WEBIMAGEM, São Paulo (SP), Brazil; and Doctoral Student in Evidence-based Health Program, Universidade Federal de São Paulo (UNIFESP), São Paulo (SP), Brazil.; II MD, MSc. Endocrinologist and Professor of Physiology and Medical Clinic, Centro Universitário Lusíada (UNILUS), Santos (SP), Brazil; and Doctoral Student in Evidence-based Health Program, Universidade Federal de São Paulo (UNIFESP), São Paulo (SP), Brazil.; III MD, PhD. Afliated Professor, Department of Neurology, Universidade Federal de São Paulo (UNIFESP), São Paulo (SP), Brazil.; IV MD, PhD. Supervising Professor, Evidence-Based Health Postgraduate Program, Universidade Federal de São Paulo (UNIFESP), São Paulo (SP), Brazil.; V PT, PhD. Associate Professor, Department of Human Movement Sciences, and Advisor, Evidence-Based Health Postgraduate Program, Universidade Federal de São Paulo (UNIFESP), São Paulo (SP), Brazil.

**Keywords:** Fractures, bone, Radiation dosage, Tomography, X-ray computed, Low-dose CT, Standard-dose CT CT scan, X-ray, ALARA, Detection rate

## Abstract

**BACKGROUND:**

Computed tomography (CT) accounts for 13% of all radiological examinations in the United States and 40-70% of the radiation that patients receive. Even with the advent of magnetic resonance imaging (MRI), CT continues to be the gold standard for diagnosing bone fractures. There is uncertainty as to whether CT with a low radiation dose has a fracture detection rate similar to that of standard-dose CT.

**OBJECTIVE:**

To determine the detection rate of low-dose radiation CT and standard-dose radiation CT for fractures, in patients with suspected fractures.

**DESIGN AND SETTING:**

Systematic review of comparative studies on diagnostic accuracy within the evidence-based health program at a federal university in São Paulo (SP), Brazil.

**METHODS:**

We searched the electronic databases Cochrane Library, MEDLINE, EMBASE and LILACS up to June 29, 2020, for studies evaluating the detection rates of low-dose CT and standard-dose CT for diagnosing bone fractures. The Research Triangle Institute (RTI) item bank tool was used for methodological quality evaluation.

**RESULTS:**

The fracture detection rate according to the number of bones evaluated, using CT with low-dose radiation was 20.3%, while with standard-dose radiation it was 19.2%, and the difference between the methods was not significant. The fracture detection rate according to the number of patients, using CT with low-dose radiation was 56.0%, while with standard-dose radiation it was 58.7%, and this difference between the methods was not significant, either.

**CONCLUSION:**

CT with low-dose radiation presented detection rates similar to those of CT with standard-dose radiation, regardless of the bones evaluated.

**REGISTRATION NUMBER:**

CRD42019148491 at the PROSPERO database.

## INTRODUCTION

Ionizing radiation such as X-rays is a singular form of energy that surmounts the binding energy of electrons that orbit atoms and molecules.^1^ In biological material exposed to X-rays, the most common consequential scenario is that this creates hydroxyl radicals from interactions between X-rays and water molecules. These radicals, in turn, interact with deoxyribonucleic acid (DNA) to cause breakage of bonds or damage to the base.^1^ Thus, mutations, chromosomal translocations and fusions between genes can occur, which in some cases may lead to cancer.^1^

All X-ray-based imaging methods have the characteristic in common of a trade-off between image quality and radiation dose, since all forms of ionizing radiation can damage tissues.^2^ In patients undergoing radiographic and/or tomographic monitoring, the patient’s exposure to radiation needs to be considered, and this poses a challenge to radiologists regarding dose reduction.^3,4^

The main concern in diagnostic imaging is that a stochastic lesion of radiation-induced cancer could develop, which can occur with any radiation dose.^5,6,7^ Conversely, deterministic effects occur only when the threshold has been exceeded and, above that, the incidence and severity of the injury increase with the radiation dose.^6,7^ It also needs to be taken into account that the pediatric population is 10 times more sensitive to radiation than adults.^8^

To date, no safe dose of ionizing radiation, below which there is no risk of cell damage and subsequent risk of cancer, has been established.^9,10^ However, it has been estimated in the United States that about 1.5%-2.0% of all malignancies can be attributed to radiation from computed tomography (CT) scans.^1,6^ Taking all imaging examinations into account, this proportion ranges from 0.6% to 3.6%.^11^ The risk of cancer increases by 0.01% for each mSv emitted in imaging tests.^12^

Thus, there is a growing awareness of the need to use the lowest possible radiation dose level that is capable of providing appropriate diagnostic information, also known as the ALARA principle (As Low As Reasonably Achievable).^9,11,12,13,14,15,16,17,18,19,20,21,22,23,24^

CT is the gold standard for diagnosing fractures,^10,25,26,27^ characterizing them in greater detail, identifying hidden fractures and showing incomplete union.^27,28^ In musculoskeletal radiology, low-dose CT has shown good results in studies with pre and postoperative scoliosis evaluations, as well as in diagnosing lytic injuries and fractures in patients with multiple myeloma.^5,29,30^ However, when metallic components are present in the bones studied, standard-dose CT scans have better image quality, with fewer artifacts, than low-dose CT scans.^31^

## OBJECTIVES

The aim of this study was to determine the detection rates of computed tomography with low radiation dose and computed tomography with standard radiation dose for fractures, independent of the bone suspected, in patients with suspected fractures.

## METHODS

### Study model

The study model followed the guidelines for systematic reviews of diagnostic accuracy studies, in the Cochrane Diagnostic Reviewer’s Handbook version 5.1.

### Inclusion criteria

The search of the literature was performed in accordance with the guidelines for Preferred Reporting Items for Systematic Reviews and Meta-Analyses (PRISMA). Studies evaluating the diagnostic accuracy and detection rates of fractures in patients with suspicion of fractures, evaluated using low-dose CT and standard-dose CT were included regardless of publication status and regardless of severity and time of disease. We did not put any restrictions on patient age, origin, language or publication status of the study. There was no exclusion regarding population size or patient age. In cases of missing information, the authors were contacted by email.

### Participants

The participants in this study were men and women of all ages with suspected bone fractures who underwent low-dose CT or standard-dose CT.

### Selection of studies and data extraction

The studies selected were those that were potentially eligible for inclusion in terms of relevant articles or abstracts from reference journals. Two authors performed independent selections for eligibility. In cases of disagreement, a third author was consulted. Data extraction was performed using a standardized form.

### Evaluation of methodological quality

Eligible studies with a control group were evaluated using the QUADAS 2 tool (Quality Assessment of Diagnostic Accuracy Studies).^32^ In all eligible studies, the RTI Item Bank questionnaire was used. This is a tool that focuses on evaluation of biases and precision).^33,34^

All forest plots were made using the Review Manager software (RevMan), version 5.3, in order to obtain sensitivity and specificity values and the respective 95% confidence intervals (CI). We expressed dichotomous data as odds ratios (OR) with a 95% CI and continuous data as mean differences (MDs) with 95% CI. The study was approved by our institutional review board, under the approval number 7184070819, dated October 2, 2019. The review was approved by the PROSPERO database. No funding or support was provided for this study.

### Research methods for choosing studies

A thorough systematic search of the literature was performed in June 2020, in the PubMed, EMBASE, Cochrane Library and LILACS online scientific publication databases, for all original-language publications. The search was conducted using the medical subject headings (MeSH). The MeSH terms used included the following: fractures, bone; radiation dosage; tomography, X-ray computed. The reference lists of the studies included and the main reviews on the subject were also evaluated. Manual searches were also carried out in the lists of references. The full search strategy is presented in **Table 1**.

**Table 1:** Search strategies used in each of the databases

Database	Search strategy
Cochrane Library	#1: MeSH descriptor: [Fractures, Bone] explode all trees. #2: MeSH descriptor: [Radiation Dosage] explode all trees. #3: MeSH descriptor: [Tomography, X-Ray Computed] explode all trees. #4: #1 AND #2 AND #3
MEDLINE	#1: “Fractures, Bone”[MeSH] OR (Broken Bones) OR (Bone, Broken) OR (Bones, Broken) OR (Broken Bone) OR (Bone Fractures) OR (Bone Fracture) OR (Fracture, Bone) OR (Spiral Fractures) OR (Fracture, Spiral) OR (Fractures, Spiral) OR (Spiral Fracture) OR (Torsion Fractures) OR (Fracture, Torsion) OR (Fractures, Torsion) OR (Torsion Fracture) #2: “Radiation Dosage”[MeSH] OR (Dosages, Radiation) OR (Radiation Dosages) OR (Dosage, Radiation) OR (Sievert Units) OR (Units, Sievert) OR (Sv Radiation Dose Equivalent) OR (Gray Units) OR (Units, Gray) OR (Gy Radiation) #3: “Tomography, X-Ray Computed”[MeSH] OR (X-Ray Computed Tomography) OR (Tomography, X-Ray Computerized) OR (Tomography, X Ray Computerized) OR (Computed X Ray Tomography) OR (X-Ray Computer Assisted Tomography) OR (X Ray Computer Assisted Tomography) OR (Tomography, X-Ray Computer Assisted) OR (Tomography, X Ray Computer Assisted) OR (Computerized Tomography, X Ray) OR (Computerized Tomography, X-Ray) OR (X-Ray Computerized Tomography) OR (CT X Ray) OR (CT X Rays) OR (X Ray, CT) OR (X Rays, CT) OR (Tomodensitometry) OR (Tomography, X Ray Computed) OR (X Ray Tomography, Computed) OR (X-Ray Tomography, Computed) OR (Computed X-Ray Tomography) OR (Tomographies, Computed X-Ray) OR (Tomography, Computed X-Ray) OR (Tomography, Xray Computed) OR (Computed Tomography, Xray) OR (Xray Computed Tomography) OR (CAT Scan, X Ray) OR (CAT Scan, X-Ray) OR (CAT Scans, X-Ray) OR (Scan, X-Ray CAT) OR (Scans, X-Ray CAT) OR (X-Ray CAT Scan) OR (X-Ray CAT Scans) OR (Tomography, Transmission Computed) OR (Computed Tomography, Transmission) OR (Transmission Computed Tomography) OR (CT Scan, X-Ray) OR (CT Scan, X Ray) OR (CT Scans, X-Ray) OR (Scan, X-Ray CT) OR (Scans, X-Ray CT) OR (X-Ray CT Scan) OR (X-Ray CT Scans) OR (Computed Tomography, X-Ray) OR (Computed Tomography, X Ray) OR (X Ray Computerized Tomography) OR (Cine-CT) OR (Cine CT) OR (Electron Beam Computed Tomography) OR (Electron Beam Tomography) OR (Beam Tomography, Electron) OR (Tomography, Electron Beam) OR (Tomography, X-Ray Computerized Axial) OR (Tomography, X Ray Computerized Axial) OR (X-Ray Computerized Axial Tomography) OR (X Ray Computerized Axial Tomography) #4: #1 AND #2 AND #3
EMBASE	#1: (‘fracture’/exp OR ‘bone cement fracture’ OR ‘bone fracture’ OR ‘closed fracture’ OR ‘fracture’ OR ‘fractures’ OR ‘fractures, bone’ OR ‘fractures, closed’ OR ‘skeleton fracture’ OR ‘unstable fracture’) #2: (‘radiation dose’/exp OR ‘dose rate, radiation’ OR ‘dose, radiation’ OR ‘radiation dosage’ OR ‘radiation dose’ OR ‘radiation dose absorption’ OR ‘radiation dose output’) #3: (‘x-ray computed tomography’/exp OR ‘ct scan’ OR ‘ct scanning’ OR ‘tomography, x-ray computed’ OR ‘x-ray computed tomography’) #4: #1 AND #2 AND #3
LILACS	#1: mh: “Fraturas Ósseas” OR (Fractures, Bone) OR (Fracturas Óseas) OR (Fratura) OR (Fraturas) OR (Fraturas de Ossos) OR (mh:C26.404) #2: “Dose de Radiação” OR (Radiation Dosage) OR (Dosis de Radiación) OR (Dosage, Radiation) OR (Gray Units) OR (Gy Radiation) OR (Sv Radiation Dose Equivalent) OR (Dosages, Radiation) OR (Radiation Dosages) OR (Units, Gray) OR (Units, Sievert) OR (Sievert Units) OR (mh: E05.799.513) OR (mh: G01.750.740) OR (mh: N06.850.810.250) OR (mh: SP8.473.654.412.062.116.157) #3: mh:”Tomografia Computadorizada por Raios X” OR (Tomography, X-Ray Computed) OR (Tomografía Computarizada por Rayos X) OR (TAC por Raios X) OR (Tomografia por Raios X Computadorizada) OR (Tomografia Axial Computadorizada por Raios X) OR (TC por Raios X) OR (Tomografia Computadorizada por Transmissão) OR (Tomografia Computadorizada por Transmissão de Raios X) OR (Tomografia Computadorizada Dinâmica) OR (Cine-TC) OR (Tomodensitometria) OR (Tomografia Computadorizada de Feixe de Elétrons) OR (Tomografia de Feixe de Elétrons) OR (Tomografia Computadorizada) OR (mh: E01.370.350.350.810) OR (mh: E01.370.350.600.350.700.810) OR (mh: E01.370.350.700.700.810) OR (mh: E01.370.350.700.810.810) OR (mh: E01.370.350.825.810.810) #4: #1 AND #2 AND #3

## RESULTS

### Studies selected

The search for this systematic review yielded 468 studies using the following MeSH terms: fractures, bone; radiation dosage; tomography, X-ray computed.

**Figure 1:**
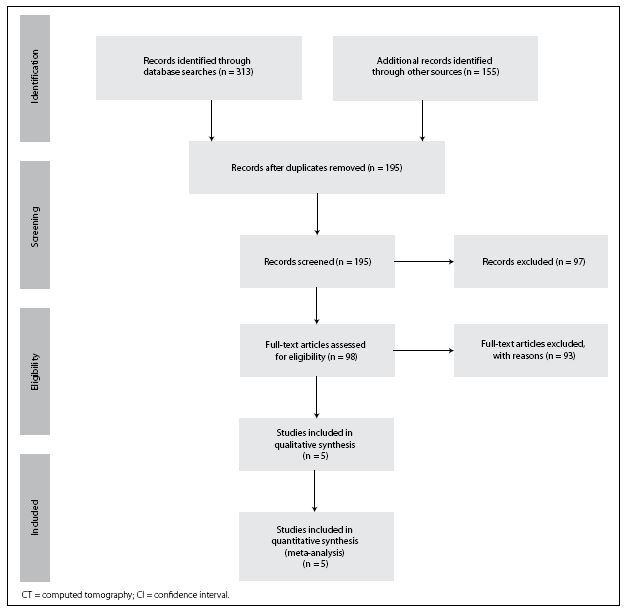
Summary of the study selection process.

There were no studies in which low-dose CT and standard-dose CT were performed on the same patient. Also, no study had a control group. Therefore, it was not possible to assess accuracy, and only the detection rate could be evaluated in the meta-analysis. A total of five studies fulfilled the inclusion criteria and were included in qualitative analysis (**Figure 1**).^16,17,18,35,36^ Two studies did not provide all the data.^16,35^ Konda et al. was not used because it did not have the necessary blinding for inclusion in this systematic review.^28^

### Analysis on the studies

Lee et al. conducted two studies: one published in 2017^17^ and another in 2018.^18^ In both, the period between January and September 2016 was assessed. However, these studies were performed using different devices, with 64 channels and 320 channels, respectively. In Lee et al.,^18^ there were evaluations by two doctors: one from the emergency department and one radiologist. For the statistical evaluation, we use the data from the radiologist because this specialist has the responsibility for issuing reports.

Four studies reported the numbers of patients evaluated and how many had fractures.^17,18,35,36^ Four studies reported the numbers of bones evaluated and how many had fractures.^16,17,18,36^ There was no study with a control group.

All the studies reported that the reduction in the radiation dosage of the CT scans was around 50%. The CT devices, bones evaluated and radiation dosages in the studies reviewed are shown in **Table 2**.^16,17,18,35,36^

**Table 2:** Radiation doses used in computed tomography in each of the studies reviewed

Study	CT device	Bone evaluated	Low-dose CT radiation	Standard-dose CT radiation
**Jin et al.^16^**	**64 MDCT**	**Rib**	1.24 mSv	5.75 mSv
**Lee et al.^17^**	**64 MDCT**	**Lumbar vertebra**	2.1 mSv	4.9 mSv
**Lee et al.^18^**	**320 MDCT**	**Lumbar vertebra**	2.1 mSv	5.4 mSv
**Mulkens et al.^35^**	**6 MDCT**	**Cervical vertebra**	1.57 mSv	3.75 mSv
	**16 MDCT**	**Cervical vertebra**	1.37 mSv	3.57 mSv
		**Ankle**	0.8 mSv	1.4 mSv
**Yi et al.^36^**	**64 MDCT**	**Pelvis**	3.9 mSv	7.4 mSv
	**Shoulder**	2.9 mSv	5.8 mSv
		**Wrist**	0.7 mSv	1.2 mSv

CT = computed tomography; MDCT = multi-detector computed tomography.

### Detection rate in relation to number of bones

**Figure 2:**
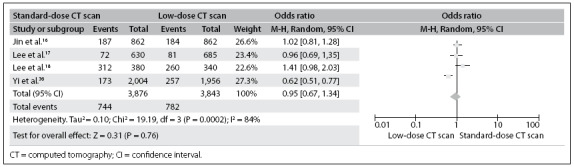
Forest plot: comparison demonstrating that there was no significant difference between low-dose CT and standard-dose CT regarding the detection rate for bone fractures.

Bone evaluations were provided and cited with regard to each method, in four studies: Jin et al.,^16^ Lee et al.,^17^ Lee et al.^18^ and Yi et al.^36^ A total of 7719 bones were evaluated. Out of the 3876 bones evaluated by means of standard-dose CT, 744 had fractures: a detection rate of 19.2%. Out of the 3,843 bones evaluated by means of low-dose CT, 782 showed fractures: a detection rate of 20.3%. All of this information is shown in **Figure 2**.

### Detection rate in relation to number of patients

**Figure 3:**
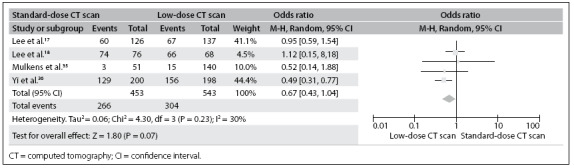
Forest plot: comparison demonstrating that there was no significant difference between low-dose CT and standard-dose CT regarding the detection rate for patients with fractures.

Patient assessments were provided and cited with regard to each method, in four studies: Lee et al.,^17^ Lee et al.,^18^ Mulkens et al.^35^ and Yi et al.^36^ A total of 996 patients were evaluated. Out of the 453 patients assessed by means of standard-dose CT, 266 had fractures: a detection rate of 58.7%. Out of the 543 patients evaluated by means of low-dose CT, 304 had fractures: a detection rate of 56.0%. All of this information is shown in **Figure 3**.

## DISCUSSION

The detection rate of low-dose CT in relation to evaluation of the number of fractured bones was 20.3%, while standarddose CT yielded a rate of 19.2%. The difference between the methods was not significant. The detection rate of low-dose CT in relation to evaluation of the number of patients with fractures was 56.0%, while standard-dose CT yielded a rate of 58.7%. Here too, the difference between the methods was not significant.

A separate assessment on joints, done in only a single study, showed a similar conclusion. Yi et al.^36^ demonstrated that fractures of the bones of the shoulder, pelvis, ankle and wrist had a similar detection rate through both low-dose CT and standard-dose CT, in terms of the evaluations on both the number of patients and the number of bones.

Lee et al.^18^ demonstrated good reproducibility among the evaluators, including between those from different specialties (a doctor in the emergency department and a radiologist). All the evaluators analyzed all the CT scans, in terms of both the number of patients and the number of bones. The detection rate for the emergency room physician was 80% in the bone assessments using standard-dose CT whereas with low-dose CT it was 73%. The detection rate for the radiologist was 82% in the bone assessments using standard-dose CT and 76% using low-dose CT. The detection rates in the evaluation according to patients was exactly the same for the two professionals: 97% with both standard-dose CT and low-dose CT.

Mulkens et al.^35^ assessed the quality of the images in low-dose CT compared with the quality in standard-dose CT. In analysis on the images done by different evaluators, it was found that although low-dose CT had lower image quality than standard-dose CT, the examination with low-dose CT preserved sufficient quality for accurate assessment of fractures. This study also demonstrated good reproducibility among the evaluators with regard to diagnosing fractures, as shown by Lee et al.^18^

Trauma is responsible for 19% of all fractures, and the incidence of these fractures also increases with age. Brazil, for example, leads the world statistics on traffic accidents, which are the predominant cause of trauma in this country.^37,38^ Thus, high numbers of fractures occur in Brazil, which requires large numbers of CT scans. Reduction of the radiation dose from CT scans in Brazil is valuable and important because this will diminish costs.

CT accounts for 13% of all radiological examinations in the United States and between 40% and 70% of the radiation that patients receive.^39,40,41^ It also needs to be taken into account that CT is responsible for the greatest exposure to radiation among trauma patients, since they frequently undergo CT scans.^7,9,15,22,42,43^ Although bones are only minimally affected by radiation, the bone marrow is the most radiosensitive organ in the body.^36^ Although CT is responsible for 40-70% of patients’ radiation doses,^39,40,41^ this percentage goes up to 97.5% in the case of hospitalized patients.^12^ In the pediatric population, the effective dose can be up to three times higher than in the adult population.^24^ The risk of developing cancer later in life is more powerfully predicted when effective doses of 5.6 mSv for the lumbar spine and 10.0 mSv for the whole dorsal spine are administered through CT, measured by means of radiography.^1,10,44^

Given that, so far, no feasible safe dose of ionizing radiation that does not present a risk of cell damage and consequently cancer has been determined,^9^ there is great interest in reducing radiation levels while maintaining the rate of fracture detection. This is even more so in the pediatric population, given that reducing the doses administered to children reduces the incidence of cancer decades after exposure. In 2007, four million CT scans were performed among children in the United States.^1^

CT can be performed with much lower doses of radiation than the standard radiation dose, despite the consequent increase in image noise and reduced image quality.^5,45,46,47^ It can even be done using the same radiation dose as in radiography.^30,48^ It seems to be particularly advantageous to indicate a reduced radiation protocol for CT on the extremities, because the area scanned is smaller than that of other regions of the human body, like the abdomen, for example.^49^

Moreover, it needs to be borne in mind that multislice CT tubes have a production life of around 800,000 slice and their average cost is 30,000 pounds (approximately 41,667.00 US dollars or 227,334.00 reais).^50^ If the radiation dose were to be halved, the useful life of the CT tube would be increased fourfold, thereby giving rise to important savings.^50^ In this regard, it also needs to be remembered that the number of CT scans performed is constantly increasing, year by year. In the United States, 70 million CT scans were performed in 2014, which was 20 times more than had been documented in 1980.^5^

The following methods can be used to reduce the patient’s radiation dose received through computed tomography:

- Reducing the milliampere-second setting: if the milliam-pere-second value is reduced by 50%, the radiation dose will be reduced by the same amount.^51^
- Increasing the pitch: the radiation dose is inversely proportional to the pitch when all other factors are kept constant.^51^
- Changing the milliamp setting according to the patient’s size: the milliamp-second value can be reduced proportionally with smaller sizes of patients.^51^
- Reducing the x-ray beam energy (kilovolt peak): reducing the beam energy results in a reduced radiation dose when all other factors are kept constant.^51^
- Model-based iterative reconstruction: this provides lower image noise and fewer artifacts; it has been designed to complement other dose-reduction methods while preserving diagnostic image quality.^52^
- Deep learning: this can distinguish noise from signal in CT images and, consequently, can boost signal while diminishing noise.^53^
- Machine-learning algorithms, as a subfield of artificial intelligence: different types of machine learning (linear regression, regression trees, bagged regression trees, Gaussian process regression, support vector machine (SVM) regression or neural networks) can reduce the radiation dosage, to adapt to new circumstances and identify and rate standards.^4^

Within the scope of public health interest, the importance of reduction of this radiation dose is in relation to the following:

- Reduction of long-term incidence of malignancies.
- Reduced spending on high-cost medications and procedures for malignant neoplasms, i.e. chemotherapy, radiotherapy, surgery, hospitalization, etc.
- Increasing the population’s quality of life.
- Reduced expenditure on CT tubes.

These changes would give rise to significant savings. They would enable reallocation of funds to areas that need more attention. Thus, this is a matter of enormous administrative relevance since, in addition to the savings already mentioned, new investments would cease to be necessary (exchange of devices, purchase of software and relocation of devices), given that only adjustments to the regulation of CT examination protocols are needed.^51^

In addition, in cases of patients with diseases that require CT monitoring, low-dose CT scans provide the possibility of shorter time intervals between examinations, thus making it possible to adjust the treatment when necessary and, hence, making it possible to avoid worsening of the disease.^54^

The present findings have some implications for future research. Low-dose CT was shown here to maintain the bone fracture detection rate and was previously shown to be effective for evaluation of pulmonary nodules^55,56^ and lithiasis in the urinary tract.^57^ In the latter, moreover, ultra-low-dose CT is already being used.^57,58^ Therefore, low-dose CT should begin to be evaluated for assessment of other structures, such as the appendix, pancreas and sinuses, among others.

Even with the advent of MRI, CT remains the gold standard for diagnosing bone fractures.^25,26,27^ Therefore, regarding evaluation of bone fractures, we believe that further studies are needed to assess the use of ultra-low-dose CT, which so far has only been analyzed by Konda et al.^28^

Ultra-low-dose CT uses a radiation dose similar to that of radiography and, consequently, further reduces the incidence of malignant neoplasms caused by standard-dose CT. Today, standard-dose CT is the cause of 1.5-2% of cases of malignant neo-plasms.^1,6^ This proportion is higher among children under 15 years old,^1,12,28^ and even more so among children younger than 5 years.^6^ Moreover, use of ultra-low-dose CT implies lower spending on medications and CT tubes. It would lead to increased quality of life for this population, over the long term.

However, as quoted by Lee,^19,39^ only 9%-16% of doctors are aware of the risk of malignant neoplasms caused by radiation. Furthermore, 75% of radiologists and on-call staff in emergency departments underestimate the radiation dose of CT, and 91% of emergency room doctors do not know that CT increases the risk of cancer throughout life. Added to this is the fact that more than 90% of patients are not informed about the dangers of radiation before they undergo CT.^19,39^ Therefore, it is extremely necessary to inform both healthcare professionals and patients about the risks of radiation and the ways in which its use in CT can be improved.

## CONCLUSION

According to the results from this systematic review and meta-analysis, it can be suggested that, in evaluating trauma victims (cases due to falls, traffic accidents, etc.) and for patients undergoing tomographic monitoring of fractures, low-dose CT should be used within clinical practice. This will reduce the radiation dose delivered to patients while maintaining the rate of fracture detection, in addition to reducing costs. Through this, it will be possible to maintain the quality of fracture diagnosis, while still avoiding complications of misdiagnosis, such as chronic arthritis, painful non-union or osteonecrosis. A decrease in CT radiation exposure is required, but image quality needs to be maintained for diagnostic accuracy.

It should be taken into account that, in our review, studies using multislice computed tomography devices with between 6 and 320 channels were evaluated, as there were no studies on other devices (helical or multislice with fewer channels) of sufficient quality for their inclusion. Evaluation of low-dose CT in patients with metallic structures was not possible since all the studies examined had excluded patients presenting metallic components (nails, screws, prostheses, etc.), from their selection of patients.
